# Complete genome of a 2014 isolate of peste des petits ruminants virus from Ethiopia

**DOI:** 10.1128/MRA.00242-23

**Published:** 2023-07-18

**Authors:** Simon King, Michael D. Baron, Menbere Kidane, Fasil Aklilu, Vivek Kapur, Catherine M. Herzog, Carrie Batten

**Affiliations:** 1 The Pirbright Institute, Woking, Surrey, United Kingdom; 2 Animal Health Institute, Sebeta, Ethiopia; 3 Center for Infectious Disease Dynamics, Pennsylvania State University, University Park, Pennsylvania, USA; Katholieke Universiteit Leuven, Leuven, Belgium

**Keywords:** peste des petits ruminants virus, Ethiopia, full-genome sequencing, phylogenetics

## Abstract

This report describes the complete genome sequence of a peste des petits ruminants virus (PPRV) isolate from Ethiopia in 2014. The strain (PPRV/Ethiopia/Habru/2014), which showed a normal virulence and relatively low morbidity in the field, belongs to the North African subclade of Lineage IV.

## ANNOUNCEMENT

Peste des petits ruminants (PPR) is a highly contagious disease affecting sheep and goats, caused by peste des petits ruminants virus (PPRV) of the genus *Morbillivirus* in the family *Paramyxoviridae* (1). PPRV is widely distributed through large parts of Africa, the Middle East, and Asia, posing an increasing threat to poor livestock keepers, primarily in developing countries (2 - 5). While there is an increasing number of PPRV full-genome sequences in databases, information on the number of cell culture passages prior to sequencing or pathogenicity associated with the viruses sequenced is generally lacking.

In 2014, samples were taken from a goat displaying typical clinical signs of PPR; the animal was part of a village flock in the district of Habru, Ethiopia (6). Morbidity rates among sheep and goats in this flock were 39.7% (48/121) and 16.4% (64/390), respectively, while case fatalities were 18.8% (9/48) and 53.1% (34/64) (6). PPRV (designated PPRV/Ethiopia/Habru/2014) was isolated in CHS-20 cells (7) from a pooled nasal/ocular swab with buccal debris, and further passaged once in CHS-20 cells and up to five times in Vero-dog-SLAM (VDS) cells (8). The third and fourth passages from VDS cells were selected for sequencing.

Nucleic acid was extracted from 200 µL of cell culture supernatant using the MagMAX Core Nucleic Acid purification kit on a KingFisher Flex automated extraction platform (both ThermoFisher Scientific, Waltham, MA). Libraries were prepared from total RNA using the Trio RNA-Seq kit (Tecan, Männedorf, Switzerland) and underwent paired-end sequencing (2 × 150 bp) on an Illumina MiSeq using v2 reagents (Illumina, San Diego, CA).

Sanger sequencing was used to determine parts of the M-F intergenic region not determined by next-generation sequencing (NGS). This region was amplified in two fragments using primers designed from the Illumina data (Table 1). Double-stranded cDNA was synthesized using SuperScript III First-Strand Synthesis System (ThermoFisher Scientific), followed by NEBNext Ultra II Non-Directional Second Strand Synthesis module (New England Biolabs, Ipswich, MA). PCR was performed with KAPA HiFi HotStart ReadyMix (Roche, Basel, Switzerland), and purified products were sequenced bi-directionally on a 3730xl DNA Analyzer (Applied Biosystems, Waltham, MA).

**TABLE 1:** Primer sequences for amplification of the M-F intergenic region

Primer	Genome position	Sequence (5′−3′)
4701Fwd	4701–4720	AACACGCCCAAGGCCAGAAG
5180Rev	5180–5160	GGTTGGATTCCCTGCCGTTTG
5041Fwd	5041–5060	TGAGAGACCCCAAGGAGAAC
5588Rev	5588–5571	AATCTGGCACGCAACAGC

A total of 292,050 and 196,212 reads were generated, with 124,730 and 35,158 reads mapping to the reference for the third and fourth passages, respectively. Next-generation sequencing (NGS) data sets were analyzed as previously described (9), using bowtie2 (10) (v2.3.4.2) and bwa mem (11) (v0.7.15) for the search, bcftools (12) (v1.6) to call the consensus, and PPRV/Ethiopia/2010 (GenBank accession KJ867541) as the reference genome. Sanger sequencing data were assembled with the NGS-derived consensus using SeqMan Pro, v17.0.2 (DNASTAR, Inc).

The complete genome has a total length of 15,948 nucleotides (GC content 47.9%). The 5′ and 3′ UTRs were determined using the NGS data. There was an average read depth of 939× and 272× per base for the third and fourth passages, respectively. No difference in sequence was found between the two passages.

**Fig 1:**
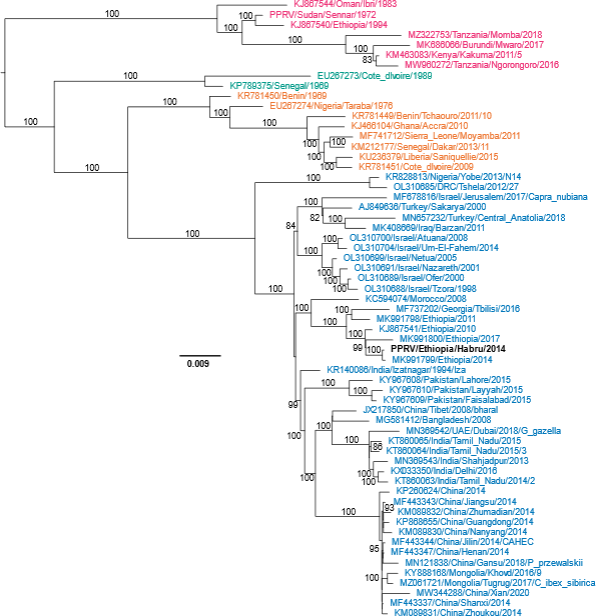
Phylogenetic analysis of PPRV/Ethiopia/Habru/2014. The full-genome sequences (bar the error-prone GC-rich M-F intergenic untranslated region) of a restricted set of virus genomes [most informative 60 sequences (13)] were added to that of PPRV/Ethiopia/Habru/2014 (black text) and the best tree calculated using RAxML (v8.2.12) (14). The genetic distances were calculated by maximum likelihood under the General Time-Reversible model with a modeled gamma-distributed variation in mutation rates to allow for variation in allowed mutation rate at different codon positions and in coding vs non-coding sequences. Standard bootstrap support for branches was calculated using the same program and is shown in the figure for all branches with support >80%. The scale bar is in units of nucleotide substitutions per site. Lineage I (green), Lineage II (orange), Lineage III (magenta), and Lineage IV (blue).

Phylogenetic comparison of the complete sequence [bar the M-F intergenic region (13)] to those of other PPRV genomes (Fig. 1) showed that the virus belongs to Lineage IV. PPRV/Ethiopia/Habru/2014 clustered with other recent Ethiopia PPRV genomes (2010–2017), as well as that from PPRV/Morocco/2008 and PPRV/Georgia/2016, suggesting that the outbreak in Georgia may have been caused by a virus imported from North Africa, and not from neighboring Turkey; all other available Turkish isolates are clustered in a separate subclade of Lineage IV.

## Data Availability

The genome of PPRV/Ethiopia/Habru/2014 has been deposited in GenBank under accession number ON110960. The raw sequence data were deposited in the NCBI Sequence Read Archive under BioProject accession number PRJNA944445.
